# No neuronal loss, but alterations of the GDNF system in asymptomatic diverticulosis

**DOI:** 10.1371/journal.pone.0171416

**Published:** 2017-02-02

**Authors:** Martina Barrenschee, Thilo Wedel, Christina Lange, Ines Hohmeier, François Cossais, Michael Ebsen, Ilka Vogel, Martina Böttner

**Affiliations:** 1 Institute of Anatomy, Kiel University, Kiel, Germany; 2 Department of Pathology, Städtisches Krankenhaus Kiel, Kiel, Germany; 3 Department of Surgery, Städtisches Krankenhaus Kiel, Kiel, Germany; University of Texas Medical Branch, UNITED STATES

## Abstract

**Background:**

Glial cell line-derived neurotrophic factor (GDNF) is a potent neurotrophic factor known to promote the survival and maintenance of neurons not only in the developing but also in the adult enteric nervous system. As diverticular disease (DD) is associated with reduced myenteric neurons, alterations of the GDNF system were studied in asymptomatic diverticulosis (diverticulosis) and DD.

**Methods:**

Morphometric analysis for quantifying myenteric ganglia and neurons were assessed in colonic full-thickness sections of patients with diverticulosis and controls. Samples of tunica muscularis (TM) and laser-microdissected myenteric ganglia from patients with diverticulosis, DD and controls were analyzed for mRNA expression levels of GDNF, GFRA1, and RET by RT-qPCR. Myenteric protein expression of both receptors was quantified by fluorescence-immunohistochemistry of patients with diverticulosis, DD, and controls.

**Results:**

Although no myenteric morphometric alterations were found in patients with diverticulosis, GDNF, GFRA1 and RET mRNA expression was down-regulated in the TM of patients with diverticulosis as well as DD. Furthermore GFRA1 and RET myenteric plexus mRNA expression of patients with diverticulosis and DD was down-regulated, whereas GDNF remained unaltered. Myenteric immunoreactivity of the receptors GFRα1 and RET was decreased in both asymptomatic diverticulosis and DD patients.

**Conclusion:**

Our data provide evidence for an impaired GDNF system at gene and protein level not only in DD but also during early stages of diverticula formation. Thus, the results strengthen the idea of a disturbed GDNF-responsiveness as contributive factor for a primary enteric neuropathy involved in the pathogenesis and disturbed intestinal motility observed in DD.

## Introduction

The formation of diverticula in the colon is a common anatomical alteration that is characterized by multiple mucosal/submucosal herniations (pseudo-diverticula) through outpouchings in the muscle layer at anatomical weak points e.g. sites of vascular perforation [1]. About 10–15% of patients develop clinically significant and symptomatic diverticulosis, leading to the so called ‘diverticular disease’ (DD) whereas others remain asymptomatic (named asymptomatic diverticulosis). DD is the 5^th^ most important gastrointestinal disease in western countries, with a considerable burden for the healthcare system [2]. In spite of its high prevalence and the socioeconomic impact, the pathogenesis of DD is still discussed controversially and considered to be multifactorial [3]. As traditional risk factors for the formation of colonic diverticula increasing age, low-fibre diet, and connective tissue alterations have been identified [4, 5]. More recently, evidence rises for an enteric neuropathy in DD, characterized by reduced nerve cells (oligo-neuronal hypoganglionosis), decreased intramuscular nerve fibers and altered neurochemical coding [6–8]. Since DD is associated with abnormal intestinal motility patterns [9, 10] it is suggested that the disease could be associated with and possibly triggered by an underlying enteric neuropathy [11].

Although a loss of enteric neurons represents a common histopathological phenotype within the spectrum of gastrointestinal neuromuscular pathology (GINMP) [12], the reason for the reduced ganglionic neuronal content observed in DD remains unclear. Recent data from our group gives evidence for an involvement of neurotrophic factors such as glial cell-line derived neurotrophic factor (GDNF) in DD. GDNF is not only well established as a potent neurotrophic factor for a variety of neuronal cell populations in the central and peripheral nervous system [13] but also for the enteric nervous system (ENS). It is known to promote the survival and maintenance in the developing and also in the adult ENS [14–16]. In patients with DD, we recently found that GDNF and its corresponding receptors GDNF family receptor alpha 1 (GFRα1) and Rearranged during transfection (RET) were significantly downregulated in the tunica muscularis [17]. We furthermore identified the dominant source of receptor and ligand expression in myenteric plexus and the tunica muscularis, respectively [18].

However, both morphometric analysis [7] with respect to consensus-based recommendations of colonic innervation disorders [19] and alteration of GDNF and GDNF receptor mRNA expression were only investigated in patients with DD [17] and data from patients with asymptomatic diverticulosis are still lacking. We therefore raised the question whether the GDNF system might be altered in earlier stages of diverticular disease formation. Thus, we performed a consensus-based morphometric analysis of the myenteric plexus and investigated the expression levels of GDNF and its receptors GFRα1 and RET in the tunica muscularis and myenteric plexus of patients with diverticulosis, DD, and controls at gene and protein level.

## Material and methods

### Tissue source

#### Control group and patients with diverticulosis

Segments of sigmoid colon were obtained from patients, who underwent partial colectomy for non-obstructive colorectal carcinoma (n = 12, 5 females, 7 males, mean age: 72 years). Patients with non-inflamed pseudo-diverticula-affected sigmoid colon as incidental finding in the control group were collected as the diverticulosis group (n = 11, 5 females, 6 males, mean age: 72 years). Anorectal evacuation and colonic motility disorders were previously excluded. Full-thickness specimens were harvested at safe distance (> 5 cm) from the tumor and immediately transferred from the operating room to the laboratory for tissue processing.

#### Patients with DD

Segments of sigmoid colon were obtained from patients (n = 13, 6 females, 7 males, mean age: 60 years), who underwent sigmoid resection or left hemicolectomy for symptomatic DD. Patients were operated after two or more attacks of diverticulitis by elective surgery. Full-thickness specimens were harvested from sites adjacent to colonic diverticula. Diverticula-containing areas displaying an altered anatomy of the colonic wall due to transmural mucosal/submucosal outpouchings of signs of inflammation and fibrotic scaring were excluded from tissue sampling. The specimens were immediately transferred from the operating room to the laboratory for tissue processing. The study of human tissue received approval from the Local Ethics Committee of the Faculty of Medicine, Kiel University, Germany (B299/07). Participants provided their written or verbal informed consent to participate in this study and the Ethics Committee approved this consent procedure.

### Tissue processing and conventional immunohistochemistry

After surgical removal all specimens were transferred into PBS (phosphate-buffered saline, pH 7.2) at 37°C to allow adaption and further dissection. Full-thickness rectangular tissue blocks (30 mm x 10 mm) were pinned out flat on a cork plate by fine needles without artificial stretching nor shortening thereby preserving the original size. The longer border of the tissue block was orientated perpendicular to the gut axis and corresponded to the cutting surface for histologic sections, so that myocytes of the circular muscle layer were cut along their longitudinal axis. After fixation (4% paraformaldehyde in PBS) for 24 h and dehydration tissue blocks were transferred into paraffin wax and cut in sections (6 μm) for immunohistochemistry.

Immunoreactive signals were visualized using the avidin-biotin-complex system (VECTASTAIN Elite ABC Kit, Vector Laboratories, Burlingame, USA). Briefly, sections were incubated with 3% hydrogen peroxide to block endogenous peroxidase activity, rinsed in TBS-buffer (TRIS-buffered saline; 10 mM Tris, 50 mM NaCl, pH 7.4) and pretreated with citrate buffer (pH 6.0, 95°C water bath, 20 min). Thereafter, samples were incubated overnight with a monoclonal mouse-anti-RET antibody (Ret01 clone 3F8; 1:200, IMGENEX, San Diego, USA), a polyclonal rabbit-anti-GFRα1 antibody (1:1000, antibodies-online.com, Aachen, Germany) or a mouse-anti-HuC/D (1:500, Molecular Probes, Invitrogen, USA) diluted in antibody diluent (Invitrogen, Karlsruhe, Germany). Sections were incubated for 45 min with biotinylated goat anti-mouse IgG (1:400, DAKO, Hamburg, Germany) for sections incubated with mouse-anti-RET or biotinylated goat anti-rabbit IgG (1:400, DAKO, Hamburg, Germany) for sections incubated with rabbit-anti-GFRα1. After washing three times with TBS, sections were incubated for 45 min with ABC conjugated with horseradish peroxidase. 3, 3’-diaminobenzidine (DAKO, Hamburg, Germany) was used as substrate chromogen. Sections were counterstained with Meyer´s hematoxylin. Omission of the primary or secondary antibody served as negative controls.

### Fluorescence-immunohistochemistry

Paraffin embedded tissue sections of control, diverticulosis or DD specimens were pre-treated with citrate buffer (pH 6.0, 95°C water bath) for 25 min followed by overnight incubation with either mouse-anti-RET (1:500, Imgenex, San Diego, USA) or rabbit-anti-GFRα1 (1:500, antibodies-online.com, Aachen, Germany) diluted in antibody diluent (Invitrogen, Karlsruhe, Germany) as primary antibodies. After washing with TBS, sections were incubated with either goat anti-rabbit AlexaFluor488 antibody or the goat-anti-mouse AlexaFluor488 antibody, diluted in antibody diluent (1:250, Invitrogen, Karlsruhe, Germany) as secondary antibodies for 2 hours at room temperature. To correct for unspecific background, (e.g. as a result of unspecific binding of the secondary antibody) fluorescence-immunohistochemistry of blank specimens were performed by processing sections from any control, diverticulosis or DD specimen without primary antibodies. Finally, all sections were stained with DAPI (Roche, Mannheim, Germany) to visualize cellular nuclei.

### Quantitation of fluorescence-imunnohistochemical signals

All image acquisitions, processing and analyses are performed under careful attention of the ethical guidelines for the appropriate use and manipulation of scientific images [20].

#### Image acquisition

Fluorescence immunohistochemical signals of n = 8 myenteric ganglia were recorded from each specimen of the control, diverticulosis and DD group (n = 8, respectively, as part of the original sample size. Control specimens: 5 females, 3 males, mean age: 75; patients with diverticulosis: 3 females, 5 males, mean age: 71, patients with DD: 4 females, 3 males, mean age 54). In addition, fluorescence immunohistochemical signals of n = 8 ganglia of every associated blank specimen (immunohistochemistry of specimen without primary antibody) were determined, respectively. Images were captured with the same settings (400x magnification, 90 ms exposure time for both antibodies, 5 ms exposure time for DAPI).

All recordings were performed using a light microscope (Axiovert 200 M, Zeiss, Germany) coupled to a digital camera (AxioCam MR3 (monochrome), Zeiss, Germany). The software program Axiovision (version 4.7, Zeiss, Germany) was used for displaying, managing and storing the generated pictures. The images are saved in the AxioVision ZVI image format and exported as 16-bit tif-format without any modifications for processing and analysis in ImageJ.

#### Image processing

Image processing were carried out in ImageJ [21]. Pictures were transformed to 8-bit and scaled. Correction of image defects caused by uneven illumination was performed using the background correction plugin from Terry Wu available at the ImageJ website (http://rsbweb.nih.gov/ij/plugins/) with iteration of 3 and radius of 6 ptx.

#### Image analysis

Each myenteric ganglion was marked and the mean grey value (integrated density/ μm^2^ ganglionic tissue) was determined. N = 8 ganglia per specimen were analysed, and the mean of these eight mean grey values were calculated for each specimen. Background correction was calculated for each specimen using the formula: Corrected mean grey value = mean grey value (target specimen)—mean grey value (blank specimen). Data were normalized to the experimental control and presented as fold increase of mean grey value.

### Morphometry of the myenteric plexus in patients with diverticulosis

As described before [7] and in line with the consensus-based recommendations on histopathological reporting for gastrointestinal neuromuscular pathologies [19] quantification of myenteric ganglia and nerve cells were performed for each of the ganglionated myenteric nerve plexus referred to the intestinal length measured in each specimen (ca. 30 mm) and extrapolated to 100 mm intestinal length to allow comparison of data. Myenteric ganglia were identified by groups of HuC/D immunoreactive neuronal somata surrounded by glial cells. Neurons were recorded based on anti-HuC/D labelled, haematoxylin counterstained full-thickness sections and characterized by a dark-red brownish nucleus and a light-red granular pericaryon. HuC/D immunoreactive cellular fragments without a discernible nucleus were excluded from the recordings for stereologic reasons to avoid overestimation of the neuronal number within a given section.

Area and number of ganglia and the neuronal number per ganglia were recorded. The following variables were calculated for statistical comparison: (i) ganglionic area, (ii) ganglionic number per intestinal length reflecting ganglionic density, (iii) neuronal number per ganglion and (iv) neuronal number per intestinal length reflecting neuronal density.

### Laser Capture Microdissection (LCM)

As described previously [22] full-thickness biopsies of the colonic wall were immediately frozen in isopentane and stored at -70°C until use. Cryosections (14 μm) were placed on membrane-coated slides (polyethylene naphtalate, 1 μm, Carl Zeiss MicroImaging GmbH, Göttingen, Germany) and regions of interest were visualized by ultra-rapid (ca. 30 s) staining with cresyl violet according to manufacturer´s instructions (P.A.L.M. RNA Handling Protocols, Zeiss MicroImaging, Göttingen, Germany). Myenteric ganglia were identified by inverse light microscopy (Axiovert, Zeiss, Jena, Germany), excised by LCM and collected by laser pressure catapulting (P.A.L.M. Microlaser Technologies, Bernried, Germany) in the cap of 0.5 ml reaction tubes. From each sample 2 mm^2^ ganglionic tissue was collected, immediately dissolved in 200 μl RNA lysis buffer (PEQLAB, Erlangen, Germany) and stored at -70°C.

### RNA extraction and reverse transcription

Extraction of total RNA from myenteric ganglia was performed using the NucleoSpin® RNA XS Kit (Machery-Nagel, Düren, Germany) according to the manufacturer´s instructions. RNA was eluted in an endvolume of 15 μl H_2_O. Prior to reverse transcription, contaminating genomic DNA was digested for 15 min at room temperature using 1.5 U of DNAse I (Sigma-Aldrich, Munich, Germany). Reverse transcription was carried out in a total volume of 30 μl containing 375 ng random hexamer primers (GE Healthcare, Freiburg, Germany), 0.5 mM dNTPs (Promega, Mannheim, Germany), 0.01 M DTT, 1 x reaction buffer, and 150 U Superscript II Reverse Transcriptase (Invitrogen, Karlsruhe, Germany). The annealing, elongation, and denaturation steps were carried out at 25°C for 10 min, at 42°C for 50 min and at 70°C for 15 min, respectively.

### Real-time quantitative PCR

Real-time quantitative PCR (qPCR) reactions were performed in 96 well plates in duplicate reactions. Each reaction (20μl) contained 2 μl of total cDNA, 900 nM primers, 225 nM hybridization probe and 1 x qPCR Master Mix Plus (Eurogentec, Cologne, Germany). qPCR product accumulation was monitored by an ABI Prism 7500 fast Real-Time PCR System (TaqMan, Thermo Fisher Scientific, Waltham, U.S.A.) over 50 cycles. Each cycle contained a denaturation phase of 15 s at 95°C and a hybridization/elongation phase of 1 min at 60°C. Primers and probes are listed below. GDNF: Forward primer: 5´-tgaaaccaaggaggaactgatttt-3´, reverse primer: 5´-gtcactcaccagccttctatttctg-3´, probe: 5´-tactgcagcggctcttgcgatgcag-3´; GFRA1: forward primer: 5´-tcgggcaatacacacctctgt-3´, reverse primer: 5´-cttggaggagcagccattga-3´, probe: 5´-tgaaaaagaaggtctcggtgcttcc-3´; RET: forward primer: 5´-aaggagatggcaaagggatcac-3´, reverse primer: 5´-ttgatgtcttgggtctccacaa-3´, probe: 5´-aggaacttctccacctgctctccc -3´; Hypoxanthine-guanine phosphoribosyltransferase HPRT (house-keeping gene): forward primer: 5´-tgaacgtcttgctcgagatgtg-3´, reverse primer: 5´-ccagcaggtcagcaaagaattt-3´, probe: 5´-tgggaggccatcacattgtagcc-3´. The data were normalized to expression levels of the housekeeping gene HPRT that did not differ significantly between the tissue sources and analysed (data not shown). Data were always presented as mean ± SEM.

### Statistics

As count data do not fulfill the requirements for normally distributed data, morphometric data were carried out by using non-parametric Mann–Whitney U-tests (Prism^**TM**^, GraphPad, Sand Diego, CA, USA) for two independent samples with p < 0.05 considered as significant. Significant outlier was calculated with Grubb’s test (Prism^**TM**^, GraphPad, Sand Diego, CA, USA) and removed from the analysis. Data are shown as median ± interquartiles and graphically presented by box-whisker plots indicating the median (horizontal line), 50% of values (box) and 99% of values (whiskers).

PCR and fluorescence quantitation data were analysed by student’s t-test (Prism^TM^, GraphPad, San Diego, CA, USA) followed by post hoc test according to the false-discovery rate procedure, using R 2.13.1 [23]. Differences were considered significant if p < 0.05 and displayed as *, whereas ** displays p < 0.01 vs control and *** = p < 0.001 vs control. Results are expressed as mean ± SEM.

## Results

### Morphometric analysis of the myenteric plexus in patients with diverticulosis

**Fig 1:**
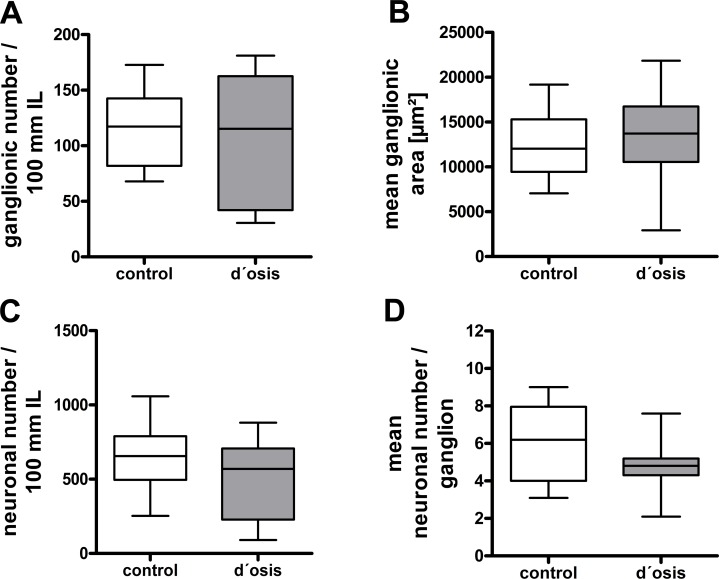
Morphometric analysis of the myenteric plexus in patients with diverticulosis compared to controls. Statistical comparison of ganglionic density (A) and mean ganglionic area (B) in patients with diverticulosis (d’osis) and controls revealed no significant differences as was neuronal density (C) and mean neuronal number (D). Data are graphically presented by box-whisker plots indicating the median (horizontal line), 50% of values (box) and 99% of values (whiskers).

Myenteric ganglionic number per 100 mm intestinal length did not differ significantly between controls (117.4 ± 70.88) and patients with diverticulosis (115.3 ± 120.6) (Fig 1A) as was the median of ganglionic area (controls 12026 ± 5857 μm; diverticulosis: 14322 ± 8618 μm)(Fig 1B). Also, the neuronal number per 100 mm intestinal length did not exhibit any significant alteration between controls (664.2 ± 469.9) and patients with diverticulosis (569.5 ± 478.5) (Fig 1C). The median neuronal number per ganglion exhibited no statistically significant reduction in patients with diverticulosis (4.8 ± 0.9) compared to controls (6.2 ± 3.95) (Fig 1D).

### Gene expression of GDNF and its corresponding receptors RET and GFRA1 in the tunica muscularis patients with diverticulosis, DD, and controls

**Fig 2:**
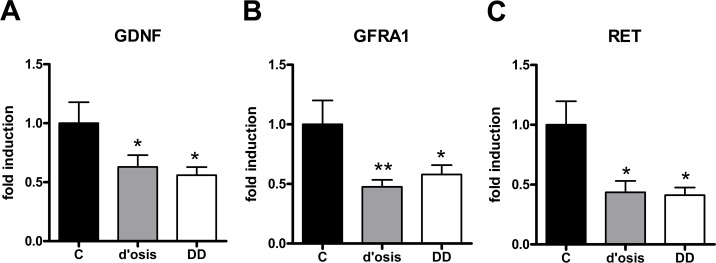
mRNA expression of GDNF and its corresponding receptors RET and GFRA1 in the colonic tunica muscularis of patients with diverticulosis, DD, and controls. mRNA expression of GDNF (A), RET (B) and GFRA1 (C) is significantly decreased in both patients with diverticulosis (d’osis) and diverticular disease (DD), when compared to controls (c). mRNA levels are determined by qPCR, results of control group compared to DD was published before [17]. Expression of target genes is normalized to mRNA expression of the house-keeping gene HPRT. Data are shown as mean +/- SEM, n = 9–12 per experimental group, *p<0.05 vs. control, **p<0.001 vs. control.

To determine the regulation of the GDNF system in patients with diverticulosis and DD compared to controls, expression levels of GDNF and its corresponding receptors GFRA1 and RET were monitored via RT-qPCR. Analysis of the tunica muscularis revealed that GDNF mRNA expression was significantly down-regulated in both patients with diverticulosis (62% ± 10% of control values) and DD (55% ± 7% of control values) (Fig 2A). Furthermore the mRNA expression of the corresponding receptor GFRA1 dropped down to 48% ± 6% of control values in patients with diverticulosis and to 58% ± 8% of control values in DD (Fig 2B). The mRNA expression of RET exhibited the strongest down-regulation with a drop-down to 43% ± 10% of control values in patients with diverticulosis and 41% ± 7% of control values in patients with DD.

### Site-specific gene expression of GDNF and its corresponding receptors RET and GFRA1 in patients with diverticulosis, DD, and controls

**Fig 3:**
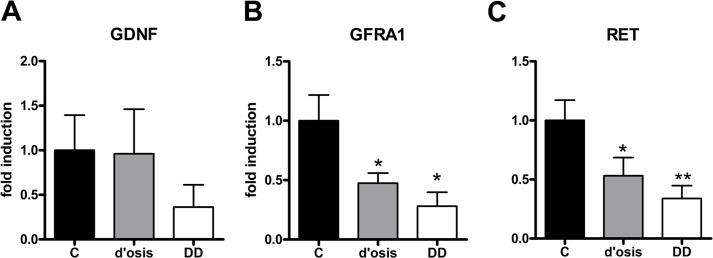
Myenteric mRNA expression of GDNF and its corresponding receptors RET and GFRA1 in the colon of patients with diverticulosis, DD, and controls. Analysis of site-specific mRNA expression profiles in microdissected myenteric ganglia reveals no alteration in the mRNA expression of GDNF (A) but a significant decrease of the GDNF receptors GFRA1 (B), and RET (C) in both patients with diverticulosis (d’osis) and diverticular disease (DD), when compared to controls (c). mRNA levels are determined by qPCR, expression of target genes is normalized to mRNA expression of the housekeeping gene HPRT. Data are shown as mean +/- SEM, n = 10–14 per experimental group, *p<0.05 vs. control, **p<0.001 vs. control.

Since enteric muscle layers and neuronal tissue vary in mRNA expression levels of GDNF and its corresponding receptors, we performed site-specific mRNA expression analysis of RET, GFRA1 and GDNF of RNA isolated from myenteric ganglia of patients with diverticulosis and DD (Fig 3). Whereas the myenteric expression level of GDNF did not differ significantly in patients with diverticulosis and DD when compared to controls (Fig 3A), the myenteric expression of GFRA1 significantly decreased to 47% ± 8% of control values in patients with diverticulosis and to 28% ± 11% of control values in patients with DD (Fig 3B). Myenteric expression of RET dropped down significantly to 53% ± 15% of control values in patients with diverticulosis and to 33% ± 10% of control values in patients with DD, respectively (Fig 3C).

### Immunohistochemistry of RET and GFRα1 in colonic myenteric ganglia of patients with diverticulosis, DD and controls

**Fig 4:**
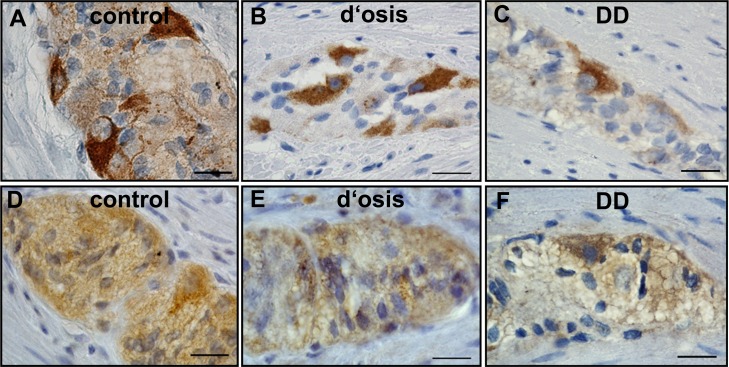
Immunohistochemistry of RET and GFRα1 in colonic myenteric ganglia of patients with diverticulosis, DD, and controls. Myenteric immunopositive signals of RET in controls exhibited robust staining in neuronal somata and weaker signals in the surrounding neuropil (A). In patients with diverticulosis (B), the robust neuronal staining was slightly decreased, whereas the surrounding neuropil exhibited a clear reduction in immunopositive signals, when compared to controls. In patients with DD (C), not only the immunopositive RET signal of the neuropil is clearly decreased, but also the neuronal staining loses intensity. Myenteric immunopositive signals of GFRα1 in controls exhibited granular staining being confined to neuronal somata and a faint staining of the neuropil (D), which was weaker in patients with diverticulosis (E). In patients with DD some neurons exhibited strong GFRα1 positive signals (arrow), whereas others displayed only weak GFRα1 positive signals (arrowhead). The neuropil exhibited only weak immunopositive signals, when compared to controls (F). Bars = 20 μm

As myenteric plexus are known to be the main source of GDNF receptor expression [18], immunopositive signals of RET and GFRα1 were investigated in colonic myenteric ganglia of patients with diverticulosis and DD (Fig 4). Controls exhibited robust immunopositive RET staining in neuronal somata and weaker signals in the surrounding neuropil, whereas GFRα1 staining was granular being confined to neuronal somata with faint staining of the neuropil (Fig 4A and 4D). In patients with diverticulosis the robust neuronal staining of RET was slightly decreased, whereas the surrounding neuropil exhibited a strong reduction in RET immunopositive signals, when compared to controls (Fig 4A). Also myenteric ganglia of GFRα1 exhibited weaker immunopositive signals in diverticulosis in both, neurons and the neuropil (Fig 4E). In patients with DD, RET positive signals were strongly decreased in the neuropil, with additional loss of staining in some, but not all myenteric neurons (Fig 4C). Also GFRα1 positive signals were strongly decreased in the myenteric neuropil in patents with DD (Fig 4F), and also in some (Fig 4F, arrowhead) but not all (Fig 4F, arrow) myenteric neurons.

**Fig 5:**
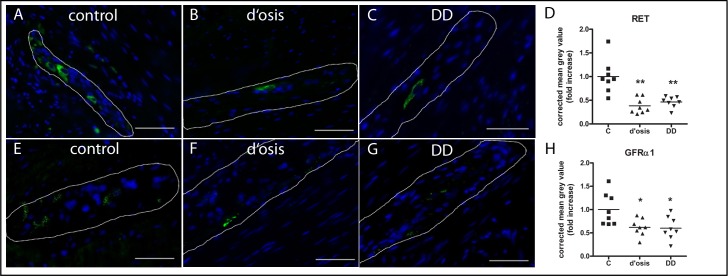
Quantitative analysis of RET and GFRα1 immunoreactivity in the myenteric plexus of patients with diverticulosis, DD, and controls. Quantitative analysis of RET immunopositive signals revealed decreased RET and GFRα1 density up to 50% in the myenteric plexus of patients with diverticulosis and DD (D), when compared to controls. Data are shown as corrected mean grey value normalized to controls presented as mean ± SEM, n = 8 per experimental group, *p<0.05 vs control, **p<0.001 vs control. Representative photographs of RET and GFRA1 immunoreactivity (green) demonstrating the myenteric plexus of controls (A, E), patients with diverticulosis (B, F) and DD (C, G). Nuclear counterstain with DAPI (blue). Bars = 50 μm

Quantitative analysis of immunopositive RET and GFRα1 signals in the myenteric plexus of patients with diverticulosis, DD and controls revealed a significant drop-down in fluorescence intensity of RET to 38% ± 5% for diverticulosis and to 46% ± 4% for DD, when compared to controls (Fig 5D). In addition, fluorescence intensity of GFRα1 is significant decreased to 61% ± 6% of the control group for diverticulosis and to 60% ± 9% for DD (Fig 5H).

## Discussion

### Morphometric changes in asymptomatic diverticulosis and DD

Our morphometric study, displayed no morphometric changes in myenteric plexus or neuron density between patients with asymptomatic diverticulosis and controls (Fig 1). In DD, morphometric alterations e.g. reduced nerve cells and ganglionic density were observed before in several studies [24, 25] and also our group reported a significant reduction of myenteric neuronal number per 100 mm intestinal length and average in neuronal number per ganglion in patients with DD [7]. However, *Iwase et*. *al* observed a reduction in number and size of myenteric ganglion cells per cm in both patients with asymptomatic diverticula and also DD [25]. But the authors did not differentiate between ganglionic cells, thus, the observed reduction might reflect glial rather than neuronal cells. Further studies have to be performed, in order to clarify the number of glial cells in diverticulosis. With respect to our data, it can be suggested, that morphologically the ENS is only disturbed in later time points of this disease perhaps after diverticula formation.

### Neurotrophic factors in asymptomatic diverticulosis and DD

#### mRNA expression

Confirmatory to recently published data [17], in this study, we observed a significant down-regulation of the neurotrophic factor ligand GDNF and its corresponding receptors RET and GFRA1 in the tunica muscularis of patients with DD compared to controls. In addition, we found both receptors and its ligand GDNF down-regulated in the tunica muscularis of patients with asymptomatic diverticulosis and this result was confirmed by site-specific gene expression experiments using RNA extracts from the myenteric plexus.

GDNF is known as an effective neurotropic factor for the ENS [15, 16], mainly expressed in the intestinal smooth muscle layers *in vivo* [18] and in smooth muscle cells *in vitro* [15]. In postnatal myenteric cell culture experiments, we could demonstrate before, that GDNF also regulates the expression of its corresponding receptors RET and GFRA1 and postulated, that the “neurotrophic factor concept” could also apply to the ENS (target-tissues produce a neurotrophic factor specifically directed toward the innervating neurons, which in turn express receptors for just these target-derived neurotropic factors for selective limitation of neuronal death) [17]. Thus, the significant impaired GDNF system in asymptomatic diverticulosis could be interpreted as primary lack of GDNF, which in turn leads to the diminished receptor mRNA expression in myenteric neurons and therefore to the neuronal cell death, observed in later stages of this disease, as demonstrated before by morphometric analysis [7].

#### Immunohistochemical quantitation

Confirmatory to mRNA expression, we observed a significant reduction in the myenteric protein expression of both GDNF receptors, GFRα1 and RET and in both patients with asymptomatic diverticulosis and DD, as quantified with fluorescence-immunohistochemistry (Fig 5). As quantitation was performed on the whole ganglia of the myenteric plexus, the main source for this reduction is still unclear. Conventional immunohistochemistry exhibited a reduction in both, the neuropil and somata of myenteric neurons for both receptors (Fig 4), which give evidences for an involvement of glia cells and neurons in the case of GFRα1. For RET, immunohistochemistry indicates only a reduction in myenteric neurons with its processes, since RET is not expressed in glia cells. However, the quantitation analysis exhibited a similar reduction in receptor expression in both diverticulosis and DD, indicating, that the process of reduced receptor expression is an early event within the pathogenesis of DD that continues during the progression of the disease. In comparison with the morphometric analysis that exhibited no reduced myenteric neurons, the data indicate that the postulated enteric neuropathy t underlying DD is an early event within the pathogenic process that is probably provoked by an impaired GDNF system.

### Pathogenetical concepts of diverticular disease

In general, the pathophysiology of DD is considered to be multifactorial and to date not adequately understood. Risk factors include e.g. increasing age, genetic predisposition, congenital connective tissue diseases, low-fiber diet and increased meat consumption [11]. However, novel concepts take into account, that patients with DD exhibit disturbed intestinal motility patterns [26, 27], morphological alteration in the ENS (oligo-neuronal hypoganglionosis) [7, 24, 25], remodeling in nerve tissue [6] as well as an impaired neuromuscular communication [28] and disturbed enteric neurotransmission [29]. These alterations in patients with DD might lead to uncoordinated contractions and high pressure, producing and triggering the formation of diverticula. Within that it is suggested, that enteric neuromuscular changes may result from typical remodeling processes after acute inflammation, since a set of neuropeptides, found to be increased after acute inflammation, were also increased in symptomatic but not inflamed DD [30]. However, a reduction in intestinal GDNF expression accompanied by a loss of enteric neurons was observed before in animal models of diabetic rats [31], and in general neurotrophic factors are expected to be up-regulated and not down-regulated during inflammatory processes, since there are evidences, that inflammatory stimuli induce GDNF expression [32]. Furthermore, it has also been shown, that GDNF levels are modified in inflammatory bowel diseases such as Crohn's disease and ulcerative colitis [33]. But within that, inflammatory stages showed induced up-regulation in GDNF concentrations in inflamed areas, whereas GDNF was down-regulated in non-inflamed tissue of patients with Crohn's disease. Thus, in the case of DD a reduced GDNF expression could also be explained as a consequence of previous inflammation that damaged the ENS or intestinal musculature. However, we demonstrated, that the down-regulation of components of the GDNF system already occurs in earlier stages of this illness, namely in asymptomatic diverticulosis, where no inflammatory events could be observed before. Thus, our results strengthen the hypothesis, that the disturbed GDNF system could be the primary trigger for the reduced neuronal number rather that inflammatory processes.

## Conclusion

To our knowledge, this is the first study addressing deficits of the GDNF system in patients with asymptomatic diverticulosis compared to DD and controls. Although no morphometric alterations of the ENS was found in patients with symptomless diverticulosis, decreased mRNA expression of GDNF and mRNA expression and fluorescence-intensity of its corresponding receptors GFRα1 and RET were found not only in DD but also in asymptomatic diverticulosis. The findings link deficits of neurotrophic factors to early stages of disease formation, and thus strengthen the hypothesis of an underlying neuropathy, contributing to the pathogenic process in DD.

## Supporting information

S1 Appendix Raw data of morphometric analysis (Table 1), mRNA expression (Tables 2 and 3) and Fluorescence-immunohistological quantification (Table 4) of controls and patients with diverticulosis and DD.
(PDF)

## Abbreviations

ENS: enteric nervous system
GDNF: Glial cell line-derived neurotropic factor
GFR**α1**: GDNF family receptor alpha 1
GI: gastrointestinal
LCM: laser microdissection
RET: rearranged during transfection
