# Supplementary material for: No neuronal loss, but alterations of the GDNF system in asymptomatic diverticulosis
Source: PLoS One. 2017 Feb 2;12(2):e0171416. doi: 10.1371/journal.pone.0171416 (PMC5289619; doi:10.1371/journal.pone.0171416)
Supplement: S1 Appendix — Raw data of morphometric analysis (Table 1), mRNA expression (Tables 2 and 3) and Fluorescence-immunohistological quantification (Table 4) of controls and patients with diverticulosis and DD. (PDF) [file pone.0171416.s001.pdf]

Table 1 Morphometric analysis of the myenteric plexus in patients with diverticulosis compared to controls

group	ganglionic number/ 100 mm IL	neuronal number/ 100 mm IL	mean ganglionic area [µm²]	mean neuronal number/ ganglion
control	114.23	789.2	19185.1	6.9
control	81.85	252.98	10457.92	3.1
control	85.32	529.01	12598.08	6.2
control	172.79	655.15	9643.47	3.8
control	117.24	537.93	7061.71	4.6
control	76.02	684.17	16394.8	9.0
control	67.9	421.92	15126.61	6.2
control	142.59	495.97	8533.58	3.5
control	262.84	2246.12	13412.64	8.5
control	117.65	673.2	9369.92	5.7
control	157.29	1058.68	11454.16	6.7
control	125	1035.71	15351.59	8.3
d'osis	111.33	498.55	15865.21	4.5
d'osis	30.51	157.64	21839.59	5.2
d'osis	121.05	490.92	9975.27	4.1
d'osis	162.6	706.69	15872.51	4.3
d'osis	33.81	228.23	14321.89	6.8
d'osis	115.25	569.49	10749.76	4.9
d'osis	173.25	880.11	12439.73	5.1
d'osis	127.39	612.44	19366.09	4.8
d'osis	181.07	786.34	13106.94	4.3
d'osis	81.07	619.93	45369.71	7.6
d'osis	42.02	90.04	2932.38	2.1

Table 2 mRNA expression of the GDNF system in the myenteric plexus in patients with controls, diverticulosis and DD, nd = not detected

group	RET mRNA Expression (LMD)	GFRa1 mRNA Expression (LMD)	GDNF mRNA Expression (LMD)
control	0.04131994	0.007181836	0.1680778
control	1.825108	2.075657425	0.5238411
control	1.622234	0.2970069	1.372876
control	0.8252952	2.468386578	2.726786
control	0.2531356	1.715427739	nd
control	1.104185	0.66368398	nd
control	0.893774	0.98184579	0.2240979
control	0.8723519	1.260126314	0.9843214
control	1.561554	0.572151638	nd
control	0.5546063	0.339421244	nd
control	0.2673429	0.829648281	nd
control	1.96939	0.789462276	nd
d'osis	1.685273	0.8517048	1.461248
d'osis	0.670348	0.6064352	nd
d'osis	0.05087082	0.2462892	nd
d'osis	0.4941561	0.4529403	0.4607964
d'osis	0.2218739	nd	nd
d'osis	0.09236435	0.2681961	nd
d'osis	0.1751649	0.1602907	nd
d'osis	0.3693939	0.5588547	nd
d'osis	nd	nd	nd
d'osis	0.7058009	0.654946	nd
d'osis	0.8493306	nd	nd
DD	0.01065727	0.03220812	0.1590113
DD	0.03024797	0.03214122	nd
DD	0.00382051	0.03586122	nd
DD	0.00253813	0.02492208	nd
DD	0.7463799	0.2676776	nd
DD	0.08980765	0.2603579	nd
DD	0.1802389	0.1121552	0.08640157
DD	0.4013637	1.514213	0.01495945
DD	0.678326	0.275203	nd
DD	0.5745164	0.07926851	0.1950886
DD	1.014684	0.08509798	1.353975

DD	nd	0.7916692	nd
DD	nd	0.1306309	nd

Table 3 mRNA expression of the GDNF system in the tunica muscularis in patients with controls, diverticulosis and DD. nd = not detected, italic = significant outlier according to Grubb's test

	GDNF mRNA Expression (TM)	GFRa1 mRNA Expression (TM)	GDNF mRNA Expression (TM)
control	0.5830443	0.4960316	0.4067635
control	1.981616	1.685882	0.6585032
control	0.6697422	0.5697907	0.2674354
control	1.134202	0.9649351	0.7995512
control	*2.57876*	2.193909	1.604654
control	0.8047863	0.6846811	1.134662
control	0.4165835	0.3544132	0.6936417
control	1.032883	0.8787373	1.368184
control	1.377142	1.171619	2.066605
d'osis	0.2061373	0.8606533	0.8779814
d'osis	0.6957677	0.5428043	0.3147465
d'osis	0.780071	0.7414924	0.9708108
d'osis	0.3157112	0.5425234	0.2792255
d'osis	1.058248	0.3188291	0.3818981
d'osis	0.7587399	0.4511946	0.7087271
d'osis	0.4094267	0.227868	0.2214093
d'osis	0.589138	0.4802336	0.1904534
d'osis	1.258476	0.2923843	0.2214093
d'osis	0.6336147	0.4802336	0.1904534
d'osis	0.7882241	0.2923843	nd
DD	1.011627	0.1753736	0.4196511
DD	0.6380218	0.5919322	0.6273152
DD	0.8715633	0.6636542	0.7538045
DD	0.6376917	0.2685949	0.1453157
DD	0.3747574	0.9003163	0.3029732
DD	0.5303421	0.6455066	0.4528993
DD	0.2678401	0.3483244	0.2711687
DD	0.5644751	0.5012158	0.6273152
DD	0.3436736	1.070663	0.3408626
DD	0.5644751	0.5390549	0.1734105
DD	0.3436736	0.6705906	*1.649767*

Table 4 Fluorescence- immunohistological quantification of RET and GFRA1 in the myenteric plexus of patients with controls, diverticulosis and DD

group	mean grey value RET	mean grey value GFRa1
control	0.9499888	0.6867377
control	1.740909	1.30342
control	0.8995702	0.6992517
control	1.042104	0.8155752
control	0.948127	0.9472668
control	0.5423943	1.244592
control	0.7095672	0.6956829
control	1.167339	1.607473
d'osis	0.6178508	0.6167984
d'osis	0.2969292	0.2963592
d'osis	0.3427994	0.5498649
d'osis	0.613646	0.6895319
d'osis	0.4696821	0.8287467
d'osis	0.2456759	0.8749174
d'osis	0.2111478	0.6163268
d'osis	0.2608431	0.4847998
DD	0.5899962	0.8450543
DD	0.4609461	0.7585432
DD	0.4953265	0.9738547
DD	0.4461281	0.5271571
DD	0.3788806	0.5054141
DD	0.538263	0.5782074
DD	0.5671014	0.2149879
DD	0.2307306	0.4092096
